# Evidence runs contrary to digestive stability predicting protein allergenicity

**DOI:** 10.1007/s11248-019-00182-x

**Published:** 2019-11-18

**Authors:** Rod A. Herman, Jason M. Roper, John X. Q. Zhang

**Affiliations:** 1 Corteva™ Agriscience, 9330 Zionsville Road, Indianapolis, IN 47968 USA; 2 Corteva™ Agriscience, P.O. Box 30, Newark, DE 19714 USA; 3 Corteva™ Agriscience, 8325 NW 62nd Avenue, Johnston, IA 50131 USA

**Keywords:** Food allergy, Digestion, Antacids, Food proteins, Risk assessment

## Abstract

A dogma has persisted for over two decades that food allergens are more stable to digestion compared with non-allergenic proteins. This belief has become enshrined in regulations designed to assess the allergenic risk of novel food proteins. While the empirical evidence accumulated over the last 20+ years has largely failed to confirm a correlation between digestive stability and the allergenic status of proteins, even those who accept this finding often assert that this shortfall is the result of faulty assay design rather than lack of causality. Here, we outline why digestive stability may not in fact correlate with allergenic potential.

## Evidence versus intuition

It is widely held that stability to pepsin under acidic conditions (e.g. in simulated gastric fluid) is correlative with a protein’s risk of causing allergy (Astwood et al. 1996; Jin et al. 2018). While the weight of evidence accumulated over the last two decades has shown that results of in-vitro gastric (and duodenal) digestion assays are not particularly useful in the allergenic risk assessment of dietary proteins (Bøgh and Madsen 2016; Herman et al. 2007), such results continue to be required by regulatory agencies that oversee the safety assessment of novel food proteins (e.g. as expressed in genetically engineered crops), and are often considered by experts in the field (Akkerdaas et al. 2018; EFSA Panel on Genetically Modified Organisms et al. 2017). In fact, the failure of the Cry9C protein expressed in genetically engineered insect-protected StarLink™ corn to be approved for food use was largely driven by its slow digestion in simulated gastric fluid (Segarra et al. 2001). Indeed, it is intuitively appealing that persistent proteins are more likely to provoke food allergy based on exposure in the gut, and it is unsurprising that such a belief often overrides the scientific evidence to the contrary. Even some experts who have concluded that results from current digestion assays are not useful in assessing allergenic potential continue to assert that more physiological assays must be useful in predicting allergenicity (Fernandez et al. 2019; Verhoeckx et al. 2019). This belief continues to be strongly promoted even though a demonstrated correlation between in-vivo human digestion of proteins and their allergenic status is lacking.

## Acid-suppressant medications and sensitization

Ostensibly, the higher frequency of food allergy among those taking acid-suppressant medications (increasing gastric pH and decreasing pepsin activity) argues for the importance of gastric digestion in allergy development and elicitation (Untersmayr and Jensen-Jarolim 2008). However, the development of allergy among this population to otherwise non-allergenic proteins (proteins not identified as causing allergy) has not been documented. Thus, the increased gastric stability of non-allergens in the presence of acid-suppressant medications has not been observed to make non-allergens allergenic. Acid-suppressant medications have only been found to make known allergens more allergenic. Further, if allergens were generally resistant to digestion in the first place, then acid-suppressant medications should have relatively less effect on their digestion compared with easily digestible proteins. As has been proposed previously, it is possible that other mechanisms explain the effect of acid-suppressant medications on the frequency of allergy to known allergens (e.g. alteration of the microbiome, a known modulator of the immune response) (Ekmay et al. 2017; Pascal et al. 2018; Robinson and Camargo 2018). The observation that acid-suppressant medications also increase the frequency of respiratory and dermal allergy argues against decreased gastric digestion as the primary mechanism by which these drugs increase allergy (Robinson and Camargo 2018). Irrespective of any mechanistic considerations, evidence is lacking that stability to gastric digestion makes non-allergens into allergens and suggests that other properties of proteins are likely driving sensitization risk (Westerhout et al. 2019).

## Gut exposure and sensitization

Evidence is accumulating for the potential sensitization to food allergens via dermal and inhalation routes of exposure, and this phenomenon may explain failure of gastric and duodenal digestion results to predict the allergenic status of proteins (Foong and Brough 2017; Herman and Ladics 2018). Furthermore, exposure in the gut is required for the normal oral tolerance process to take place that prevents food allergy to most dietary proteins, and reducing the digestive stability of an allergen can actually lessen its ability to tolerize and prevent allergy (Chinthrajah et al. 2016; Freidl et al. 2019). This required gut exposure for tolerization indicates that incomplete gastric and duodenal digestion occurs for most dietary proteins rendering them non-allergens. While it is largely unknown how the level and timing of exposure in the gut might influence the sensitization versus tolerance processes, exposing children to allergenic foods early in life is now recommended to promote oral tolerance and reduce the frequency of food allergy (Turcanu et al. 2017). Thus, it should be considered that low exposure in the gut may not prevent proteins from sensitizing individuals, and that high exposure in the gut may not correlate with sensitization risk.

## Evidence guides good risk assessment

We put forth that it is not clear that digestion results (physiological or not) will inform the allergenicity risk assessment for novel food proteins that have a low potential for exhibiting cross-reactivity (low amino acid sequence similarity) with known allergens. It is critical that any new assay developed for use in the weight of evidence for assessing the allergenic potential of novel food proteins be correlative with the allergenic status of known allergens and non-allergens and provide added value beyond bioinformatic data. Specifically, assay results should be able to distinguish allergens from non-allergens within the same protein family using a common threshold of stability across protein families; a common threshold of concern is important because novel food proteins are unlikely to belong to the relatively few protein families that contain allergens, and thus, novel food proteins will most often not have paired allergens against which results can be compared (Akkerdaas et al. 2018; Herman and Ladics 2018). While intuition is key to forming hypotheses, evidence should be paramount when accepting or rejecting hypotheses and in weighing the evidence used to evaluate health risks, including the allergenic risk of novel food proteins (Sackett and Rosenberg 1995).
